# Asthma Associations in Children Attending a Museum of Science

**DOI:** 10.3390/ijerph10094117

**Published:** 2013-09-04

**Authors:** Laura Corlin, Mark Woodin, Danny Newhide, Erika Brown, Sarah Valentina Diaz, Amy Chi, Doug Brugge

**Affiliations:** 1 Community Health Program, Tufts University School of Arts and Sciences, Medford, MA 02155, USA; E-Mails: laura.corlin@tufts.edu (L.C.); danny.newhide@gmail.com (D.N.); erikabrown1215@gmail.com (E.B.); sarah_val_diaz@yahoo.com (S.V.D.); 2 Department of Civil and Environmental Engineering, School of Engineering, Tufts University, Medford, MA 02155, USA; E-Mail: mark.woodin@tufts.edu; 3 Tufts Medical Center, Boston, MA 02111, USA; E-Mail: achi@tuftsmedicalcenter.org; 4 Department of Public Health and Community Medicine, Tufts University School of Medicine, Boston, MA 02111, USA

**Keywords:** pediatric, asthma, risk factors, respiratory, family history, allergies, medical history, environment, urban

## Abstract

We explored the relative strength of environmental and social factors associated with pediatric asthma in middle class families and considered the efficacy of recruitment for an educational study at a science museum. Eligibility criteria were having a child aged 4–12 and English fluency. Our questionnaire included information on demographics, home environment, medical history, and environmental toxicant exposures. Statistically significant associations were found for: child’s age (*t* = −2.46; *p* = 0.014), allergies (OR = 11.5; 95%CI = 5.9–22.5), maternal asthma (OR = 2.2; 95%CI = 1.2–3.9), parents’ education level (OR = 0.5; 95%CI = 0.3–0.9), family income (OR = 2.4; 95%CI = 1.1–5.5), water damage at home (OR = 2.5; 95%CI = 1.1–5.5), stuffed animals in bedroom (OR = 0.4; 95%CI = 0.2–0.7), hospitalization within a week after birth (OR = 3.2; 95%CI = 1.4–7.0), diagnosis of pneumonia (OR = 2.8; 95%CI = 1.4–5.9), and multiple colds in a year (OR = 2.9; 95%CI = 1.5–5.7). Several other associations approached statistical significance, including African American race (OR = 3.3; 95%CI = 1.0–10.7), vitamin D supplement directive (OR = 0.2; 95%CI = 0.02–1.2), mice in the home (OR = 0.5, 95%CI = 0.2–1.1), and cockroaches in the home (OR = 4.3; CI = 0.8–21.6). In logistic regression, age, parents’ education, allergies, mold allergies, hospitalization after birth, stuffed animals in the bedroom, vitamin D supplement directive, and water damage in the home were all significant independent predictors of asthma. The urban science museum was a low-resource approach to address the relative importance of risk factors in this population.

## 1. Introduction

Over 12 percent of U.S. children have been diagnosed with asthma in their lifetimes [1]. Males, children who are of non-Hispanic black race, and children in low income families are more likely to have been diagnosed with asthma [2]. While environmental factors, such as second hand smoke [3] and allergens [4,5], and physical factors, such as exercise [6] and respiratory illnesses [7], are known to exacerbate asthma attacks, less is known about each factor’s relative role in the pathogenesis of asthma. Biologic, environmental, and social factors appear to interact in priming the immune system towards an allergic or atopic response [8]. Studies have shown strong familial inheritance of asthma [9] and genome-wide association studies have identified several genetic loci with strong linkages to asthma [10]. However, genetics alone do not explain the rising incidence of asthma. Social and environmental factors such as allergen exposure [11,12,13,14] and ambient air pollution [15,16] may play a role. Additionally, prenatal exposure to tobacco [17,18,19] and maternal vitamin D status [20,21] may be implicated in the increasing development of childhood asthma. 

The set of factors that influence asthma onset is complex and may vary between different populations. While most studies to date focus on traditionally underserved or marginalized populations, fewer have studied the risk factors for asthma in predominantly middle class U.S. populations. We recruited at a museum of science, a low-resource approach for reaching our target population. Among the handful of studies that have considered asthma in these populations, exposure to allergens [22,23], psychosocial factors [24,25], and African American race [26,27,28] were associated with asthma. By comparing numerous social, medical, and environmental risk factors in a single survey, we sought evidence for their relative importance. 

## 2. Experimental Section

### 2.1. Consent/Institutional Review Board Approval

The Tufts Medical Center Institutional Review Board and Boston Museum of Science approved the study protocol as a minimal risk study. All researchers had completed human research ethics training. Recruitment and data collection were conducted within the parameters of an on-site educational research program. This approach allows scientists to collect data within the museum exhibits, educate visitors about their research topics, and work with museum educators to introduce the public to science. The parent or legal guardian was given a one page disclosure statement indicating the purpose and procedure of the study and detailing participants’ rights, including that participation was completely voluntary and that participants could withdraw at any time. Oral consent was obtained from the parent or guardian prior to the survey administration. The data collected were anonymous.

### 2.2. Recruitment

Parents and legal guardians of children aged 4–12 who were English speaking were eligible to participate. Non-English speakers were excluded because the museum is an English language venue. Children under four years of age were excluded because of the uncertainty of asthma diagnosis in this age group. Asthma status was not assessed during recruitment. Visitors to the museum could participate in the educational component without participating in data collection. Parents and legal guardians accompanying children who appeared eligible were approached in 2011 and 2012. Recruiters used a number of strategies such as approaching families whose children participated in an interactive museum display that compared the child’s height to others of their age range so that a more accurate height estimate could be obtained. Researchers would also specifically approach parents that seemed to have fewer children that they were supervising or families in which more than one adult was present to increase the likelihood that it would be feasible for the parent to participate in the short survey. A sign was posted to attract participants. No formal data were collected on the number of potential participants approached who chose not to participate or on the number of participants who came to the researchers to ask to participate but researchers noted that approximately 20–60% of parents would agree to participate when approached. Parents seemed more willing to participate if they had fewer children to watch, if their children were slightly older, or if they were given the option of moving through the exhibit with their children while the survey was being administered. 

After the initial consent process, researchers administered the survey orally to parents. Children were given an optional activity while the survey was completed. After the survey was complete, or for museum guests who chose only to participate in the educational component, researchers offered to explain the basic pathophysiology of asthma using diagrams of the lungs and bronchioles. Researchers also let the child “feel” what asthma is like by breathing through a pinched straw. Children were given stickers for participating in the educational component and/or for their parents/guardians participation in the survey. Basic composite survey findings to date were available to participants on the laptop after they completed the survey. 

The questionnaire consisted of 35 questions. Demographic data collected were sex, age, height, weight, race/ethnicity, country of birth, family income, parents’ education level, perceived neighborhood safety, and parent-reported doctor diagnosis of asthma and allergies. We sought to ask about most of the known or suspected factors associated with the development of asthma in children. Survey questions to determine children’s health factors were low birth weight, hospitalization within a week after birth, number of months breastfed as an infant, and family history of asthma or allergies. Other questions asked about environmental exposures to tobacco smoke (both pre and postnatal), pests, infectious diseases, stuffed animals, thick curtains and blankets, mold, pets, analgesics, traffic proximity, doctor recommendation to take vitamin D, and the amount of time spent with other children in daycare or in the home. 

In addition to the questions included for the main analysis, 38 participants also participated in a process survey about their experience completing the study in the Museum of Science. This survey was administered by researchers after the asthma survey to willing parents.

### 2.3. Data Management and Statistical Analysis

Data were entered into MS Excel in real time and then transferred to SPSS (Version 21) for data cleaning and analysis. The final sample size was 303 children after restricting the sample to children ages 4 through 12 and excluding two children with no response to the asthma question. We examined all variables with descriptive statistics. The primary outcome variable of interest was yes or no to doctor diagnosed asthma. Bivariate associations were assessed with χ² coefficients and odds ratios. Most variables were dichotomized for analysis of bivariate associations. Certain categorical variables were broken into several levels on the study questionnaire. For example, the association between asthma and income was assessed at three levels (income ≤$50,000, income between $50,000–100,000, and income >$100,000). Age was assessed as a continuous variable.

We built binary logistic regression models beginning with all of the variables that had significant bivariate associations. Sex and self-identification as African American were also included in the initial model even though neither variable had a significant bivariate association. Both of these variables were subsequently excluded since neither contributed to the models’ ability to accurately predict asthma status and neither was significant in the model. 

Colinearity was then assessed for the remaining variables. While several of the variables were significantly correlated with other variables, none of the associations was stronger than 0.382. However, since having a mother who had asthma, a family income below $50,000, and prior pneumonia status all were collinear with other variables in the model and none was significant, these three variables were removed from the model. Mold allergies were added to the final adjusted model despite their significant bivariate correlation with allergy status (r = 0.382, *p* < 0.0005) because they retained an independent, significant, and strong effect in the final model. Mold also had significant bivariate correlations with hospitalization within a week after birth (r = 0.156) and colds (r = 0.189). Having more than three colds in a year was taken out of the final model because it was not significant when controlling for the remaining variables. 

The final model included age, allergy status, mold allergy status, a parent with a graduate degree, a directive by a doctor to take vitamin D supplements, presence of visible water damage in the home, presence of more than 10 stuffed animals in the child’s bedroom, and hospitalization within a week after birth. Age was included as a continuous variable. We used a Hosmer and Lemeshow value of at least 0.1 as a minimum threshold for models. 

## 3. Results and Discussion

### 3.1. Results

The mean age of the 303 children in our sample was 7.8 years. Half of the children were male (50.8%). The children were predominately white (84.2%), from homes with incomes greater than $100,000 (63.6%), and had at least one parent with a graduate degree (63.8%). Almost one-fifth of the children in our convenience sample had asthma (19.5%). Demographic factors associated with asthma were age above eight years and annual family income below $50,000. Since there were only nine participants in the study with a family income of less than $25,000 and a sub-analysis showed that the same proportion of participants with a family income of less than $25,000 had asthma as participants with a family income of less than $50,000 (3/9 compared to 10/30), the results for the lower two income brackets were combined. African Americans were more likely to have asthma (41.7% of the 12 African Americans had asthma *vs.* 17.6% of whites), but the association did not quite reach significance. Children who had at least one parent who had completed a graduate level degree were less likely to have asthma. Table 1 summarizes the descriptive statistics and bivariate associations with asthma for the demographic factors. 

**Table 1:** Demographic characteristics.

	Asthmatic (%, n)	Not-Asthmatic (%, n)	*p* Value	Odds ratio [95% CI]
**Age** ^a^	8.5 (2.2)	7.7 (2.4)	**0.014**	**−2.460**
Male	52.5 (31)	50.4 (123)	0.769	0.9 [0.5–1.6]
Race/Ethnicity				
*White, not-Hispanic*	79.3 (46)	88.5 (216)	*0.063*	0.5 [0.2–1.0]
Asian American	8.8 (5)	8.6 (21)	0.968	1.0 [0.4–2.8]
Hispanic	8.6 (5)	4.9 (12)	0.271	1.8 [0.6–5.4]
*African American*	8.8 (5)	2.9 (7)	*0.055*	3.3 [1.0–10.7]
Foreign Born	10.2 (6)	6.6 (16)	0.346	1.6 [0.6–4.3]
Annual Family Income				
**≤$50,000**	18.9 (10)	9.2 (20)	**0.033**	**2.4 [1.1–5.5]**
>$50,000–100,000	24.5 (13)	26.0 (59)	0.826	0.9 [0.5–1.9]
>$100,000	56.6 (30)	65.2 (148)	0.242	0.7 [0.4–1.3]
Parents’ Education Completed				
*High School or less*	11.9 (7)	5.4 (13)	*0.073*	2.4 [0.9–6.2]
College	37.3 (22)	27.9 (67)	0.147	1.5 [0.9–2.8]
**Graduate**	50.9 (30)	66.9 (162)	**0.021**	**0.5 [0.3–0.9]**
Fear of crime	1.7 (1)	3.7 (9)	0.434	0.4 [0.1–3.6]

^a^ Age given as mean (standard deviation), *p* value, and *t* value; *p* values <0.05 are bolded, *p* values between 0.05 and 0.10, inclusive, are italicized.

Table 2 shows medical and familial factors associated with asthma in bivariate analysis. These included a biological mother with a history of asthma, hospitalization within a week after birth, allergies of all types (including plant, animal, chemical, and mold), and a history of diagnosed pneumonia or more than three colds in a year. Table 3 shows the descriptive statistics and bivariate associations with asthma for the environmental factors. Visible water damage in the home was significantly positively associated with asthma while having more than 10 stuffed animals in the bedroom and being told by a doctor that the child needed to take vitamin D supplements were significant negative associations. Non-statistically significant associations suggested that children who lived in a home with cockroaches were more likely to have asthma while children who lived in a home with mice were less likely to have asthma. 

**Table 2:** Medical and familial factors.

	Asthmatic(%, n)	Not-Asthmatic(%, n)	*p* Value	Odds ratio [95% CI]
**Mother asthmatic**	42.4 (25)	25.4 (60)	**0.010**	**2.2 [1.2–3.9]**
Birth weight <2,500 g	8.8 (5)	5.1 (12)	0.289	1.8 [0.6–5.3]
**Hospitalized after birth**	20.3 (12)	7.4 (18)	**0.003**	**3.2 [1.4–7.0]**
Breastfed	66.7 (38)	67.1 (163)	0.953	1.0 [0.5–1.8]
Overweight or Obese	41.8 (23)	34.6 (72)	0.323	1.4 [0.7–2.5]
Takes pain medication often	1.8 (1)	1.7 (4)	0.972	1.0 [0.1–9.5]
Vitamins				
*Told to take vitamin D supplements*	1.8 (1)	9.9 (24)	*0.087*	0.2 [0.02–1.2]
Never wear sunscreen	6.8 (4)	11.5 (28)	0.288	0.6 [0.2–1.7]
Always sunscreen	13.6 (8)	14.0 (34)	0.931	1.0 [0.4–2.2]
**Allergies**	74.6 (44)	20.2 (49)	**<0.001**	**11.5 [5.9–22.5]**
**Allergies-Pollen**	44.8 (26)	6.3 (15)	**<0.001**	**12.1 [5.8–25.3]**
**Allergies-Cats**	31.0 (18)	3.0 (7)	**<0.001**	**14.8 [5.8–37.7]**
**Allergies-Dogs**	20.7 (12)	2.1 (5)	**<0.001**	**12.1 [4.1–36.0]**
**Allergies-Mice**	3.4 (2)	0.4 (1)	*0.100*	8.4 [0.8–94.6]
**Allergies-Dustmites**	39.7 (23)	3.8 (9)	**<0.001**	**16.6 [7.1–38.9]**
**Allergies-Cockroach**	5.2 (3)	0 (0)	**<0.001**	NA
**Allergies-Food**	34.5 (20)	5.9 (14)	**<0.001**	**8.4 [3.9–18.0]**
*Allergies-Chemical*	5.2 (3)	1.3 (3)	*0.093*	4.3 [0.8–21.7]
**Allergies-Mold**	24.1 (14)	1.3 (3)	**<0.001**	**24.6 [6.8–89.2]**
Infectious Diseases ^a^				
Respiratory Syncitial Virus-MD	5.1 (3)	2.9 (7)	0.407	1.8 [0.4–7.1]
Respiratory Syncitial Virus-Hosp	0 (0)	0 (0)	NA	NA
**Pneumonia-MD**	27.6 (16)	12.1 (28)	**0.003**	**2.8 [1.4–5.6]**
Pneumonia-Hosp	1.7 (1)	1.7 (4)	0.997	1.0 [0.1–9.1]
Chicken Pox-MD	8.7 (5)	6.5 (15)	0.544	1.4 [0.5–4.0]
Chicken Pox-Hosp	0 (0)	0 (0)	NA	NA
**Colds-MD**	33.3 (19)	14.6 (34)	**0.001**	**2.9 [1.5–5.7]**
**Colds-Hosp**	3.5 (2)	0 (0)	**0.004**	NA
Measles-MD	0 (0)	0 (0)	NA	NA
Flu-MD	26.3 (15)	20.8 (48)	0.365	1.4 [0.7–2.7]
Flu-Hosp	1.8 (1)	0.9 (2)	0.557	2.0 [0.2–22.9]
Parasites-MD	0 (0)	0.4 (1)	0.619	NA
Parasites-Hosp	0 (0)	0 (0)	NA	NA
Hepatitis-MD	0 (0)	0 (0)	NA	NA

^a^ MD = diagnosed by a doctor, Hosp = hospitalized for disease

**Table 3:** Environmental factors.

	Asthmatic (%, n)	Not-Asthmatic (%, n)	*p* Value	Odds ratio [95% CI]
Only child	20.3 (12)	22.5 (55)	0.715	0.9 [0.4–1.8]
Have pets	64.4 (38)	64.8 (158)	0.960	1.0 [0.5–1.8]
Lived on a farm	3.4 (2)	3.7 (9)	0.908	0.9 [0.2–4.3]
Horseback	10.2 (6)	11.6 (28)	0.761	0.9 [0.3–2.2]
*Mice ever*	13.6 (8)	24.1 (58)	*0.081*	0.5 [0.2–1.1]
Current mice	10.2 (6)	11.7 (28)	0.746	0.9 [0.3–2.2]
Rats ever	0 (0)	0.83 (2)	0.482	NA
Current rats	0 (0)	0 (0)	NA	NA
*Cockroach ever*	5.1 (3)	1.2 (3)	*0.093*	4.3 [0.8–21.6]
Current cockroach	3.4 (2)	1.2 (3)	0.249	2.8 [0.5–17.1]
Carpet	62.7 (37)	55.6 (134)	0.323	1.3 [0.7–2.4]
Pillows	67.8 (40)	72.7 (176)	0.451	0.8 [0.4–1.5]
Comforter	84.7 (50)	88.5 (215)	0.433	0.7 [0.3–1.6]
Thick curtain	13.6 (8)	11.2 (27)	0.606	1.2 [0.5–2.9]
Thick blanket	28.8 (17)	38.8 (94)	0.152	0.6 [0.3–1.2]
**Stuffed Animal **	52.5 (31)	74.4 (180)	**0.001**	**0.4 [0.2–0.7]**
**Water damage **	19.3 (11)	8.9 (21)	**0.023**	**2.5 [1.1–5.5]**
Prenatal SHS	3.4 (2)	2.5 (6)	0.712	1.4 [0.3–6.9]
Postnatal SHS	8.5 (5)	7.4 (18)	0.782	1.2 [0.4–3.3]
Lived <100 m of highway as infant	5.2 (3)	8.8 (21)	0.369	0.6 [0.2–2.0]

Following the descriptive and univariate analysis, potential predictors that were associated with asthma (*p* < 0.10) were examined. In the full multivariate model, the variables we assessed for their association with asthma included age, sex, African American race, family income below $50,000, parent education level, a maternal history of asthma or allergies, hospitalization within a week after birth, allergies, previous diagnosis of pneumonia, previous diagnoses of more than three colds in a year, being told by a doctor that the child has a need for vitamin D supplements, more than 10 stuffed animals in the child’s bedroom, and visible water damage in the home (Table 4). In the final model, allergies, mold allergies, hospitalization within a week after birth, visible water damage, and age were all independent predictors of asthma diagnosis (Table 5). Both allergies and allergies to mold were included in the adjusted model because even controlling for allergic status, which increased the likelihood of an asthma diagnoses by almost 13-fold, a child with mold allergy was still seven times more likely to have asthma than a child without mold allergies. Tolerance and correlation statistics indicated that both variables independently contributed to the final R^2^ of the model. None of the other types of allergies was significantly associated with asthma after controlling for the other variables in the model. Protective factors in the adjusted model included being told by a doctor that the child needed to take vitamin D supplements, having at least 10 stuffed animals in the child’s bedroom, and having a parent with a graduate degree. For example, children who were directed to take vitamin D supplements were 96.2% less likely to have asthma. 

**Table 4:** Full model.

	Beta	SE	*p* Value	Odds ratio
Age	0.234	0.098	**0.017**	1.264
Sex	0.185	0.442	0.675	1.204
African American	1.206	0.869	0.165	3.339
Income <$50 K	0.523	0.677	0.439	1.688
Graduate degree	−0.842	0.445	0.059	0.431
Mother asthma	0.620	0.458	0.176	1.858
Hospitalized after birth	0.764	0.710	0.282	2.146
**Allergies**	2.657	0.461	**<0.001**	14.259
Pneumonia	0.057	0.543	0.917	1.058
**Colds**	1.054	0.521	**0.043**	2.869
Vitamin D	−1.912	1.269	0.132	0.148
**Stuffed animals**	−1.112	0.481	**0.021**	0.329
**Water damage**	1.159	0.583	**0.047**	3.187

**Table 5:** Final model.

	Beta	SE	*p* Value	Odds ratio
**Age**	0.185	0.088	**0.036**	1.203
**Graduate degree**	−0.816	0.404	**0.044**	0.442
**Hospitalized after birth**	1.227	0.570	**0.031**	3.410
**Allergies**	2.545	0.431	**<0.001**	12.744
**Mold Allergies**	1.957	0.841	**0.020**	7.078
**Stuffed animals**	−1.453	0.420	**0.001**	0.234
**Vitamin D**	−3.278	1.570	**0.037**	0.038
**Water damage**	1.098	0.541	**0.042**	2.998

Among the 38 respondents to the process survey, two-thirds were female and 82% were between 31 and 50 years old. Approximately half of the participants lived in Massachusetts and eight percent lived in Boston. Over three quarters of process survey respondents indicated that they had a very good or excellent experience participating in the study and 92.1% of respondents indicated that they were somewhat likely or very likely to participate in another similar research study at the Museum of Science. Almost all respondents (94.6%) thought that it was probably or definitely a good idea to conduct scientific research in a science museum setting. Respondents indicated a range of reasons for participation in the study including: to help improve the health of the public, they knew someone the research might help, curiosity about the research, wanting their child to witness their participation, and wanting to help the researchers.

### 3.2. Discussion

The Museum of Science, and particularly the Human Body Connection exhibit, provided several advantages as a recruitment site. One goal of translational research is to educate the public on cutting edge science. The inclusion of an interactive educational component seemed to be a draw for participants who likely already had some interest in learning about health and disease although no formal feedback specifically was collected. Participants reported that the experience was positive and that they saw value in conducting research in such a setting. The recruitment location had the added advantage of allowing us to study asthma in a fairly homogenous middle class population. Almost 64% of the children in our study lived in families with an annual income of over $100,000 and a similar percentage had at least one parent with a graduate degree. Previous work on asthma has typically focused on individuals of lower socioeconomic status. 

We replicated several widely reported positive associations with asthma, including measures of SES, family history of asthma, allergies, frequent colds, and mold. Fitting of a hypothesis generating study design, two of our associations were particularly interesting. First, we found an association between a physician directive to take vitamin D supplements and decreased asthma prevalence. Second, water damage and mold in the home and mold allergy appeared to be relatively important. It is worth noting that some commonly reported associations with asthma were either extremely rare in our sample (e.g., low family income, taking pain medications, ever living on a farm, prenatal tobacco smoke exposure, fear of crime) or were present, but not associated with asthma (e.g., breastfeeding, overweight or obese, pillows, carpet, and comforters). For those exposures present but not associated with asthma, our analysis could suggest that these are less important factors for middle class American children. 

There are a very limited number of studies considering asthma specifically in pediatric middle class populations [22,23,24,25,26,27,28]. Among these few studies, early life infections and parenting difficulties were associated with later asthma onset in a prospective study of middle class children with at least one asthmatic parent [25]. Consistent with previous studies, we found that asthma in middle-class children is associated with multiple, diverse factors [29,30]. In our sample, the strongest risk factors for asthma included allergies, particularly mold allergies, hospitalization within a week after birth, visible water damage in the home (also likely an indicator of mold), and increased age. The strongest protective factors were doctor recommendation for vitamin D supplements, having at least 10 stuffed animals in the child’s bedroom, and having at least one parent with a graduate degree. 

The role of vitamin D in the development of asthma has been controversial. Some epidemiological studies have associated low vitamin D intake with an increased risk of asthma [20,31,32,33,34], but results are not completely consistent and there may be a greater effect in males than females [35]. It seems likely that vitamin D has an immunoregulatory function such that a low intake of vitamin D predisposes individuals to an atopic response [31,32,36,37]. We found that children with asthma were 96.2% less likely to have been told they needed vitamin D supplements. As our question was only a proxy for vitamin D levels and we did not have data on actual vitamin D levels, the quantity of supplements taken, why the supplements were recommended, or whether they were taken at all, there is some potential for misclassification bias. Most likely, this would be random misclassification and would underestimate any possible association with asthma since we have no reason to believe that parents would be more likely to inaccurately report whether their children had been told to take vitamin D based on asthma status. Regardless, we do not know if any children had true vitamin D insufficiency and only 8% of children were told that they needed supplements, including only one child with asthma, so the results should be interpreted with caution. 

Another strong protective association in the adjusted model was the presence of at least 10 stuffed animals in the child’s bedroom. There are several possible interpretations. It could be that exposure to increased levels of dust mites, which are associated with stuffed animals promoted proper immune system development as has been suggested elsewhere [38]. However, since none of the other measures of items that collect dust in the bedroom, such as presence of carpet or thick curtains, were significantly associated with asthma, it is possible that the number of stuffed animals in a child’s bedroom is not a good approximation for dust mite exposure. We chose to ask about 10 stuffed animals to distinguish between children who had many stuffed animals in their bedroom and children who did not. It is possible that the choice of this cut point affected our findings. Conversely, parents may remove these asthma triggers after the child’s diagnosis which could explain the reduced likelihood of having stuffed animals in asthmatic children’s bedrooms. Since we only have cross sectional data, we cannot say which of these situations is most likely. The strongest risk factors in the multivariate model were not surprising. The association between asthma and allergy is well established and is linked with similar molecular pathways [39,40]. Consistent with other studies [41], mold allergies in particular were very strongly associated with asthma, even controlling for general allergic status and other factors. This finding was of particular importance in this sample because water damage in the home was also significantly associated with asthma, even after controlling for mold allergies [42]. Longitudinal cohort studies have suggested that living in homes with mold odor [13] and visible mold [43] increases the risk of future pediatric asthma onset. Hospitalization within a week after birth was also strongly associated with pediatric asthma in the adjusted model, although parents were not asked the reason for hospitalization. This finding is consistent with many other studies showing that neonatal hospitalizations for conditions such as respiratory distress [44] are important for respiratory health later in life [45,46], controlling for factors such as postnatal secondhand smoke exposure, low birth weight, and growing up in an urban environment [44]. 

It is worth noting several factors that were not found to be significantly associated with asthma in our sample. In one study considering African American and white children of similar SES, African Americans were found to have higher asthma prevalence than whites [47]. Our study had only a limited number of African American participants (nine percent of our sample) but it is possible that if we had a larger sample, there would have been a statistically higher prevalence of asthma among these children as we found a non-statistically significant trend of African American children being more likely to have asthma. However, race was not significant once variables relating to SES were controlled for, consistent with several other studies of pediatric asthma [48]. Additionally, while studies suggest associations between air quality and asthma [49,50], our population had low prevalence of exposure to second hand smoke and other indicators of poor air quality.

### 3.3. Limitations

There were several limitations in this study. We had a cross-sectional design and thus could not evaluate the temporal sequence of exposures relative to asthma diagnosis. This limitation is illustrated by the challenge of interpreting exposure to stuffed animals in the bedroom as parents could have modified the dust levels in response to pre-existing asthma in the child. All data were reported by the parent or caregiver without objective measures of exposure or asthma diagnosis. However, most of our questions were easily observed and it is likely that parents were reasonably accurate. It is possible that parents of asthmatic children were more likely to report the presence of risk factors than were parents of non-asthmatic children. There is also a risk of chance findings given the large number of associations tested, although this was intended to be an exploratory study. Representativeness of the sample is not certain given that it was a convenience sample. Some selection bias is likely since potential participants approached may have been more willing to participate if they had a family member with asthma or allergies. The prevalence of asthma in our sample is likely an overestimate of the museum population. Additionally, some selection bias could have resulted if parents and caregivers who attended the museum with fewer children or more than one adult were more likely to participate. Furthermore, our results may not be generalizable to a non-English speaking middle class population. Finally, some of the exposures, such as rats or cockroaches in the home, were quite rare in our sample limiting our ability to draw strong conclusions about the association between these variables and asthma and possibly reflecting the fact that the data were collected in a public setting where certain exposures could be underreported. Future prospective studies should consider a wide range of potential risk factors to verify which social, familial, and environmental components are of greatest importance in asthma etiology for middle class children. 

## 4. Conclusions

Our findings are preliminary in many ways, but they point to the potential for greater examination of determinants of asthma in middle class children revealing the relative importance of various risk factors. Since these children have a different set of exposures from inner-city, low-income and primarily minority children, the associations we found could give us another window into why some children develop asthma and others do not. We found the museum of science to be a viable venue from which to recruit middle class children.
